# Hybrid method to solve HP model on 3D lattice and to probe protein stability upon amino acid mutations

**DOI:** 10.1186/s12918-017-0459-4

**Published:** 2017-09-21

**Authors:** Yuzhen Guo, Fengying Tao, Zikai Wu, Yong Wang

**Affiliations:** 1 Department of Mathematics, Nanjing University of Aeronautics and Astronautics, Nanjing, 210000 People’s Republic of China; 2 National Center for Mathematics and Interdisciplinary Sciences, Academy of Mathematics and Systems Science, Chinese Academy of Sciences, Beijing, 100190 People’s Republic of China; 3 University of Chinese Academy of Sciences, Beijing, 100049 People’s Republic of China; 4 University of Shanghai for Science and Technology, Shanghai, 200433 People’s Republic of China; 5 Shanghai Key Laboratory of Intelligent Information Processing, Fudan University, Shanghai, 200433 People’s Republic of China

**Keywords:** Protein structure prediction, HP model, 3D lattice, Particle swarm optimization, Protein stability

## Abstract

**Background:**

Predicting protein structure from amino acid sequence is a prominent problem in computational biology. The long range interactions (or non-local interactions) are known as the main source of complexity for protein folding and dynamics and play the dominant role in the compact architecture. Some simple but exact model, such as HP model, captures the pain point for this difficult problem and has important implications to understand the mapping between protein sequence and structure.

**Results:**

In this paper, we formulate the biological problem into optimization model to study the hydrophobic-hydrophilic model on 3D square lattice. This is a combinatorial optimization problem and known as NP-hard. Particle swarm optimization is utilized as the heuristic framework to solve the hard problem. To avoid premature in computation, we incorporated the Tabu search strategy. In addition, a pulling strategy was designed to accelerate the convergence of algorithm based on the characteristic of native protein structure. Together a novel hybrid method combining particle swarm optimization, Tabu strategy, and pulling strategy can fold the amino acid sequences on 3D square lattice efficiently. Promising results are reported in several examples by comparing with existing methods. This allows us to use this tool to study the protein stability upon amino acid mutation on 3D lattice. In particular, we evaluate the effect of single amino acid mutation and double amino acids mutation via 3D HP lattice model and some useful insights are derived.

**Conclusion:**

We propose a novel hybrid method to combine several heuristic strategies to study HP model on 3D lattice. The results indicate that our hybrid method can predict protein structure more accurately and efficiently. Furthermore, it serves as a useful tools to probe the protein stability on 3D lattice and provides some biological insights.

## Background

Protein is the substantial basis of biological activity. The function of protein is determined by its structure which is believed to be decided by the amino acid sequence according to Anfinsen’s experiments. So the research on protein structure prediction (also called protein folding problem) is very significant and fundamental in exploring the fundamental principle to map sequence, structure, and function.

To capture the backbone of protein structure prediction, Dill and his collaborators introduced HP lattice model to simplify real world complexity in 1995 [1]. HP lattice model is an abstracted scaffold, and eventually convert the protein structure prediction problem to an optimization problem on lattice. The aim is to find the optimal structure with the lowest energy. Computationally, solving this problem is NP-hard. For this reason many researchers have been attracted to study this problem by proposing many heuristic algorithms. In recent years, for 2D HP protein folding problem, many methods have been proposed, e.g., PSO (Particle Swarm Optimization) [2], ACO (Ant Colony Algorithm) [3], ABO (Artificial Bee Colony) [4] and SOM (Self-Organizing Mapping) [5] etc.

One issue for 2D lattice model is that it’s too simplified to constrain the amino acid sequence on a 2D plane. One step forward is to fold the sequence on 3D lattice and make it a better and native approximation. So far, several algorithms have been applied for 3D HP protein structure prediction problem, such as UEGO (Universal Evolutionary Global Optimization) [6], GA (Genetic Algorithms) [7], TS (Tabu Search) [8], EA (Evolutionary Algorithm) [9] and so on. Each method has its advantage to capture some special structure in the problem. In this paper, we aim to propose a hybrid method and improve the efficiency to solve the 3D HP protein structure prediction problem.

PSO was introduced by Kennedy and Eberhart [10]. It is a swarm intelligence optimization algorithm which imitates the foraging behaviors of birds and fish. As a simple meta-heuristic, it has been used to solve optimization problem with nonlinear, non-differentiable, and multi-modal function. Originally, this algorithm was designed for solving continuous optimization problem. Here, we started from the basic PSO framework and firstly extend the algorithm to the combinatorial optimization, into which we formally formulate the HP model on 3D lattice. In addition, we improved PSO as follows: a) redefined velocity for discrete model; b) employed modified Tabu search strategy to avoid premature convergence; c) designed pulling strategy to speed up convergence.

We showed that our hybrid algorithm can predict structures of amino acid sequences with different length efficiently. With this useful tool, we simulated the effects after single amino acid mutation and double amino acids mutation, respectively. Some biological insights are obtained.

The remainder of this paper is organized as follows. Firstly, a mathematical model was established for 3D HP problem. Secondly, we explained the PSO algorithm and proposed modified Tabu search method and pulling strategy. Thirdly, the performance of our algorithm was validated. Fourthly, the amino acid mutation result was obtained and analyzed. Finally, conclusions were presented.

## Methods

### Combinatorial optimization formulation for 3D HP lattice model

In HP model, every amino acid sequence is abstracted as an alphabetic string with H (hydrophobic amino acid) and P (hydrophilic amino acid). The protein conformation is a self-avoiding path on a 2D lattice. It is assumed that the main driving forces of the formation of the tertiary structure are the interactions among hydrophobic amino acids which are adjacent on lattice but not adjacent in the sequence, denoted as H-H interactions. The free energy of a protein conformation (*X*) is expressed by the number of H-H interactions. Based on Anfinsen’s assumption [11], the configuration tends to form a core in the spatial structure shield from the surrounding solvent by hydrophilic amino acids with the minimal free energy. So the more H-H interactions, the lower the free energy. We assumed that the free energy equals to the minus number of H-H interactions. HP lattice model has been used for solving protein structure prediction problem on 2D and 3D lattices widely. In this paper, we focused on the 3D HP square lattice model.

**Fig. 1:**
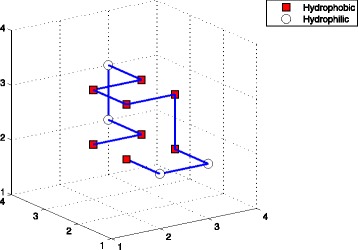
The structure of sequence (*H*_2_ *P*_2_ *H*_5_ *P*_2_ *H*) on 3D square lattice

At present, relative coordinates and space coordinates have been used to denote the protein conformation. For a sequence *S* with *L* amino acids, *X* is a string of length *L*−1 over the symbols {*r*(*ight*),*l*(*eft*),*f*(*orward*),*d*(*own*),*u*(*p*)} in relative coordinates, these five symbols reflect the relative location of contiguous amino acids on lattice. In space coordinates, *X* records the 3D coordinates of *L* amino acids, namely, *X*=(*X*(1),*X*(2)⋯*X*(*L*)) and *X*(*l*)∈*N* ^3^(*l*=1,2⋯*L*) is the coordinate of the *l* ^*th*^ amino acid. In this paper, we chose the space coordinates. For example, Fig. 1 showed a conformation with 7 H-H interactions on 3D square lattice. Its conformation was denoted as *X*=((2,3,2),(3,3,2),(3,4,2),(3,4,3),(3,3,3),(2,3,3),(2,2,3),(3,2,3),(3,2,2),(3,1,2),(2,1,2),(2,2,2)).

Based on the abstraction and minimum energy principle, we established the optimization model (OM) for protein structure prediction problem on 3D square lattice as following:

1 $$\begin{array}{*{20}l} & min\quad E(X)  \end{array} $$

2 $$\begin{array}{*{20}l} & s.t.\quad \sum_{i=1}^{I}\sum_{j=1}^{J}\sum_{k=1}^{K}x_{\text{\textit{i,j,k}}}(l)=1 \quad \quad l=1,2\cdots L  \end{array} $$

3 $$\begin{array}{*{20}l} & \quad \quad 0\leq\sum_{l=1}^{L}x_{\text{\textit{i,j,k}}}(l)\leq1 \quad \quad l=1,2\cdots L  \end{array} $$

4 $$\begin{array}{*{20}l} & \quad \quad \sum_{d=1}^{3}|X(l+1)_{d}-X(l)_{d}|\cdot\|X(l+1)-X(l)\|=1\\ & \qquad \qquad \qquad \qquad \quad \quad \quad l=1,2\cdots L-1  \end{array} $$

Here,

5 $$\begin{array}{*{20}l}{} E(X) =&-M(X)  \end{array} $$

6 $$\begin{array}{*{20}l}{} M\! =&\!\sum\limits_{i=1}^{I}\!\sum\limits_{j=1}^{J}\!\sum\limits_{k=1}^{K}\!\sum\limits_{l=1}^{L}x_{\text{\textit{i,j,k}}}(l)f(l)\!\sum\limits_{r=1}^{L}f(r)[\!x_{i,j,k+1}(r)\,+\,x_{i,j+1,k}(r)\quad\quad\\ &+x_{i+1,j,k}(r)]-h  \end{array} $$

7 $$\begin{array}{*{20}l}{} h =&\sum_{l=1}^{L-1}f(l)f(l+1)  \end{array} $$

8 $$\begin{array}{*{20}l}{} x_{\text{\textit{i,j,k}}}(l) =& \begin{cases} 1 &\text{if the \(X(l)=(i,j,k)\)}\\ 0 &\text{else} \end{cases}  \end{array} $$

9 $$\begin{array}{*{20}l}{} f(l)=& \begin{cases} 1 &\text{if the \(l^{th}\) amino acid is H}\\ 0 &\text{if the \(l^{th}\) amino acid is P} \end{cases}  \end{array} $$

Where, *E*(*X*) is the free energy of protein conformation *X*, *X*(*l*)_*d*_ is the *d* ^*th*^ component of *X*(*l*), *M*(*X*) is the number of H-H interactions in conformation *X*, *r* expresses the number of adjacent hydrophobic pairs in amino acid sequence and ∥·∥ is Hamming distance. Equations (2), (3) and (4) constrain that every amino acid occupies only one lattice point, each lattice point cannot be used more than once and adjacent amino acids in the chain occupy the adjacent points on the lattice. Equation (8) presents whether the *l* ^*th*^ amino acid occupies point (*i,j,k*). In Eq. (9), *f*(*l*) translates the *l* ^*th*^ H (or P) of the amino acid sequence into 1 (or 0).

Solving the simplified HP model is NP-complete even on two dimensional lattice. Then we have to seek help from heuristic algorithms. Particle swarm optimization, one of the stochastic algorithm, serves as a powerful approximation method.

### Hybrid algorithm

#### The basic PSO algorithm

Particle swarm optimization (PSO) is a heuristic framework that optimizes an objective function by iteratively improve a candidate solution. The motivation is to have a population of candidate particles, and move these particles around in the search-space according to simple mathematical formulae over the particle’s position and velocity. Each particle’s movement is influenced by its local best known position, but is also guided toward the best known positions in the search-space, which are updated as better positions are found by other particles. Finally it is expected to move the swarm toward the best solution. The advantage of PSO is that it makes no assumptions about the problem and can search very large spaces of candidate solutions.

In basic PSO algorithm (See Table 1), *m* particles search the optimal position simultaneously with dynamic velocity. Particle velocity is affected by iteration, own cognition, and social cognition of particle. Particularly, each particle can remember not only its own flight experience, but also the trajectories of all particles. In *n* dimensional search space, the position and velocity of the *i* ^*th*^ particle are represented as *X*_*i*_∈*R* ^*n*^ and *V*_*i*_∈*R* ^*n*^, respectively. They are updated by the following two equations:

10 $$\begin{array}{*{20}l} {} V^{t+1}_{i}&=\omega V^{t}_{i}+c_{1}r_{1}\left(P^{t}_{ib}-X^{t}_{i}\right)+c_{2}r_{2}\left(P^{t}_{gb}-X^{t}_{i}\right)  \end{array} $$

**Table 1:** The process of basic PSO algorithm

**Step 1**	To **initialize** $\{X^{0}_{i}|i=1,2\cdots m\}$ and $\{V^{0}_{i}|i=1,2\cdots m\}$;
**Step 2**	To **calculate** $E(X^{t}_{i})$, find $P^{t}_{ib}$ and $P^{t}_{gb}$ ;
**Step 3**	To **update** $X^{t}_{i}$ and $V^{t}_{i}$;
**Step 4**	To **output** *P*_*gb*_.

11 $$\begin{array}{*{20}l} {} X^{t+1}_{i}&=X^{t}_{i}+V^{t+1}_{i}  \end{array} $$

Where $P^{t}_{ib}$ and $P^{t}_{gb}$ are the best position of the *i* ^*th*^ particle and the best position of all particles in the *t* ^*th*^ iteration, respectively. Inertia weight (*ω*), self confidence (*c*_1_) and swarm confidence (*c*_2_) are input parameters, *r*_1_,*r*_2_ are two separately generated uniformly distributed random numbers in the range [0,1].

#### The modified PSO algorithm

##### Definitions

To solve the optimization model, we redefined position and velocity of PSO on 3D lattice. Particle position was orderly expressed by protein conformation (*X*). Velocity of particle was defined as a series of shift (*j*_1_,*j*_2_), which means that the $j_{1}^{th}$ component of particle position becomes the $j_{2}^{th}$ component, then the $j_{1}^{th}$ component and the $j_{2}^{th}$ component (including the $j_{2}^{th}$ component) were changed subsequently. In addition, position *X*_1_ was obtained by the sum of position *X*_2_ and a series of shift, namely *X*_1_=*X*_2_+{(*j*_*p*_,*j*_*q*_)}. For example, *V*={(2,4),(3,1)} and *X*=(*X*(1),*X*(2),*X*(3),*X*(4)), then

$$\begin{array}{@{}rcl@{}} X+V&=& (X(1),X(2),X(3),X(4))+\{(2,4),(3,1)\} \\ &=& (X(1),X(3),X(4),X(2))+\{(3,1)\} \\ &=& (X(4),X(1),X(3),X(2)). \end{array} $$

Clearly, *X*+*V* is a new position. Nevertheless, the new position may not satisfy the constraints in the OM model. An adjustment strategy is needed to ensure the new position was valid.

##### Modified Tabu search strategy

Premature convergence is one of the major difficulty to solve OM model by PSO algorithm. To further improve the modified PSO, we adopted the idea of Tabu search which was proposed by Glover [12]. This method was briefly described as follows.

Tabu search is a meta-heuristic method that maintains only one solution in the iteratively searching process. Given an initial solution *X*, the idea is to calculate and compare its neighboring solutions *N*(*X*). The best solution is chosen as candidate solution *X*_*c*_. If *X*_*c*_ is satisfied with the aspiration rule, it will replace the current solution *X* and be added to tabu list *T*_*list*_; Otherwise, the current solution *X* will be replaced by the best one *X* ^′^ (*E*(*X* ^′^)=*min*{*E*(*X*)|*X*∈*N*(*X*),*X*∉*T*_*list*_}) and *X* ^′^ will be added to *T*_*list*_. Generally, *T*_*list*_ is a first-in first-out (fifo) memory with limited length. So particles would not search the solutions which have been found for a while, simultaneously, the better solutions would not always be taboo.

Neighbourhood of solution and aspiration rule are the key components of Tabu search. In our 3D HP problem, feasible solution is a 3D self-avoiding path. It was not easy to figure out its neighboring solutions from a given solution. According to Eqs. (10) and (11), we got similar solutions by changing *r*_1_,*r*_2_ at the same iteration for the same particle in PSO, then these solutions constituted a neighbourhood. When candidate solution was better than the current solution, we would ignore whether the candidate solution was taboo or not.

##### Pulling strategy

The convergence rate of modified PSO with Tabu search strategy is not fast enough and the conformations obtained by this modified PSO may be too loose. The following strategy was designed in order to improve the algorithm.

**Fig. 2:**
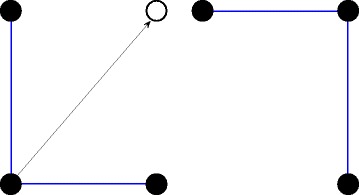
The flow of pulling strategy. Left figure is for structure before pulled. Right figure is for structure after pulled

In native protein structure, hydrophobic amino acids concentrate inside of conformation and they were surrounded by hydrophilic amino acids. If hydrophilic amino acids were pulled out of the central of protein structure, the structure will be more compact and more stable. Without changing structure’s legitimacy, this strategy was defined as pulling strategy. In order to make pulled structure to satisfy the self-avoiding constraints, only one amino acid could be pulled to its vacant diagonal position once. Figure 2 showed the move and result of one pulling.

##### Hybrid method

A novel hybrid method was proposed by combining modified PSO with modified Tabu search strategy algorithm, denoted as TPPSO^1^. Another hybrid method was taken as TPPSO^2^, which combined TPPSO^1^ with pulling strategy. Both methods employed Tabu search strategy and were applied to solve protein structure prediction problem. In TPPSO^1^ and TPPSO^2^, when *P*_*ib*_ and *P*_*gb*_ were found, *s* alternative particles would be produced by Eqs. (10) and (11) for each particle.

We selected different *r*_1_ and *r*_2_ for finding alternative particles. These alternative particles might not satisfy the constraints, therefore they should be adjusted. Then the best alternative particle would replace the previous particle and *P*_*gb*_ would be taboo in a period of time. Differently, pulling strategy has been used in TPPSO^2^, so each particle could be closer to optimal position. Table 2 showed the detailed procedures of TPPSO^2^.

**Table 2:** The algorithm outline of TPPSO ^2^

**Step 1**	To **initialize** $\{X^{0}_{i}|i=1,2\cdots m\},\{V^{0}_{i}|i=1,2\cdots m\}$ and *T*_*list*_=Ø;
**Step 2**	To **calculate** $E(X^{t}_{i})$, find $P^{t}_{ib}$ and $P^{t}_{gb}$;
**Step 3**	To **update** $\{V^{t}_{ij}|j=1,2\cdots s\}$ and $\{X^{t}_{ij}|j=1,2\cdots s\}$;
**Step 4**	To **adjust** and pull $\{X^{t}_{ij}|j=1,2\cdots s\}$ ;
**Step 5**	To **calculate** $E(X^{t}_{ic})=min\{E(X^{t}_{ij})|j=1,2\cdots s\}$;
**Step 6**	**If** $E(X^{t}_{ic})\leq E(X^{t}_{i})$ then $X^{t}_{i}=X^{t}_{ic}$;
**Step 7**	To **calculate** $E(P^{t}_{gbc})=min\{E(X^{t}_{i})|i=1,2\cdots m\}$;
**Step 8**	**If** $E(P^{t}_{gbc})< E(X^{t}_{gb})$ then $X^{t}_{i}=X^{t}_{ic},T_{list}=\text {{\O }}$;
**Step 9**	**If** $E(P^{t}_{gbc})= E(X^{t}_{gb})$ and $P^{t}_{gbc}\notin T_{list}$ then $T_{list}=T_{list}+X^{t}_{gb}, X^{t}_{gb}=X^{t}_{gbc}$;
**Step 10**	To **output** *P*_*gb*_.

## Results

### Numerical simulations

In order to test the feasibility of the hybrid algorithms (TPPSO^1^ and TPPSO^2^) and explore the properties of algorithms, we calculated two groups of amino acids sequences, respectively.

#### Simulation of sequences with 27 amino acids

We selected 11 sequences with 27 amino acids (See Table 3) which were also computed by EN [13] and hELP [14]. These sequences were used to test the performances of TPPSO^1^ (without pulling strategy) and TPPSO^2^ (with pulling strategy), respectively. In TPPSO^1^ and TPPSO^2^, the inertia weight *ω* was updated by the following formula:

12 $$ \omega=0.1-0.05\frac{Time}{Maxtime}  $$

**Table 3:** Sequences with 27 amino acids used in our study

Sequence ID	Amino acids sequence
*A*_1_	*PHPHPH*_3_ *P*_2_ *HPHP*_11_ *H*_2_ *P*
*A*_2_	*PH*_2_ *P*_10_ *H*_2_ *P*_2_ *H*_2_ *P*_2_ *HP*_2_ *HPH*
*A*_3_	*H*_4_ *P*_5_ *HP*_4_ *H*_3_ *P*_9_ *H*
*A*_4_	*H*_3_ *P*_2_ *H*_4_ *P*_3_ *HPHP*_2_ *H*_2_ *P*_2_ *HP*_3_ *H*_2_
*A*_5_	*H*_4_ *P*_4_ *HPH*_2_ *P*_3_ *H*_2_ *P*_10_
*A*_6_	*HP*_6_ *HPH*_3_ *P*_2_ *H*_2_ *P*_3_ *HP*_4_ *HPH*
*A*_7_	*HP*_2_ *HPH*_2_ *P*_3_ *HP*_5_ *HPH*_2_ *PHPHPH*_2_
*A*_8_	*HP*_11_ *HPHP*_8_ *HPH*_2_
*A*_9_	*P*_7_ *H*_3_ *P*_3_ *HPH*_2_ *P*_3_ *HP*_2_ *HP*_3_
*A*_10_	*P*_5_ *H*_2_ *PHPHPHPHP*_2_ *H*_2_ *PH*_2_ *PHP*_3_
*A*_11_	*HP*_4_ *H*_4_ *P*_2_ *HPHPH*_3_ *PHP*_2_ *H*_2_ *P*_2_ *H*

The *Time* is the circular times and *Maxtime* is the maximum number of iterations which is 3000 in our implementation. For each particle, we chose *c*_1_=*c*_2_=1, *r*_11_=*rand*(0.9,1), *r*_12_=*rand*(0.82,0.92), *r*_13_=*rand*(0.74,0.84), *r*_21_=*rand*(0.9,1), *r*_22_=*rand*(0.85,0.95), *r*_23_=*rand*(0.8,0.9) to produce three similar but not identical alternative particles. In this test, *T*_*list*_ only contained ten particles.

According to Table 4, we knows that all the sequences in Table 3 were simulated by EN, hELP, and our method TPPSO^1^ and TPPSO^2^. hELP and TPPSO^2^ can obtain the minimal free energy of every sequence, but EN and TPPSO^1^ can’t find the minimal free energies of sequence *A*_6_, *A*_7_, and *A*_11_ which are bigger. It illustrated our method can successfully predict the protein structure on 3D square lattice. The number in parentheses is the iteration number when the lowest free energy values are found. By comparing the results of hELP with TPPSO^1^ and TPPSO^2^, TPPSO^1^ is superior to hELP, and TPPSO^2^ can fold stable structures earlier than TPPSO^1^.

**Table 4:** Comparing four algorithms in eleven sequences with 27 amino acids

Sequence ID	EN	hELP	TPPSO^1^	TPPSO^2^
*A*_1_	-9	-9(18009)	-9(1983)	-9(177)
*A*_2_	-10	-10(9447)	-10(1304)	-10(439)
*A*_3_	-8	-8(1420)	-8(1249)	-8(44)
*A*_4_	-15	-15(2125)	-15(795)	-15(19)
*A*_5_	-8	-8(2877)	-8(104)	-8(61)
*A*_6_	-11	-12(2610)	-11(940)	**-12**(812)
*A*_7_	-13	-13(3967)	-12(721)	**-13**(805)
*A*_8_	-4	-4(1070)	-4(6)	-4(3)
*A*_9_	-7	-7(363)	-7(389)	-7(14)
*A*_10_	-11	-11(416)	-11(2784)	-11(83)
*A*_11_	-14	-16(285)	-14(957)	**-16**(2672)

The number in parentheses is the iteration number before the lowest free energy values are found. TPPSO^2^ can find the optimal results of all sequences. TPPSO^1^ can’t obtain the minimal free energies for sequence *A*_6_, *A*_7_, and *A*_11_ (highlighted in bold)

**Fig. 3:**
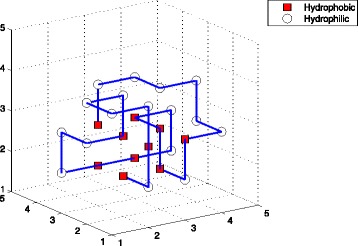
This is one of structures for sequence *A*_6_. This optimal conformation was simulated by TPPSO^2^ with 12 H-H interactions. *Squares* are for hydrophobic amino acids, and *circles* are for hydrophilic amino acids. In this structure all hydrophobic amino acids are surrounded in center. It is stable with minimal free energy

**Fig. 4:**
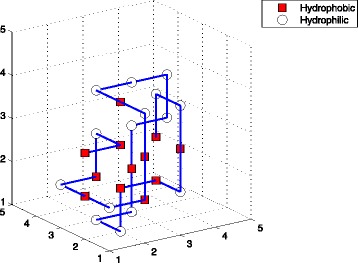
This is one of structures for sequence *A*_7_. This optimal conformation was simulated by TPPSO^2^ with 13 H-H interactions. *Squares* are for hydrophobic amino acids, and *circles* are for hydrophilic amino acids. In this structure almost all hydrophobic amino acids are surrounded in center. It is stable with minimal free energy

**Fig. 5:**
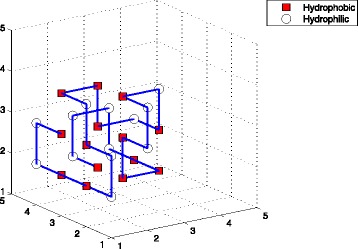
This is one of structures for sequence *A*_11_. This optimal conformation was simulated by TPPSO^2^ with 16 H-H interactions. *Squares* are for hydrophobic amino acids, and *circles* are for hydrophilic amino acids. It is stable and compact with minimal free energy

Especially, TPPSO^2^ found the lowest free energies of sequences *A*_6_, *A*_7_, and *A*_11_, while TPPSO^1^ or EN did not. So we enumerated the structures of these sequences (See Figs. 3, 4, and 5), which were computed by TPPSO^2^. It can be seen that these conformations were more native, furthermore, the hydrophobic amino acids were concentrated and surrounded by hydrophilic amino acids. Because pulling strategy of TPPSO^2^ has not been employed in TPPSO^1^, it is understandable that pulling strategy was able to accelerate the convergence of algorithm and optimize the protein structure.

**Fig. 6:**
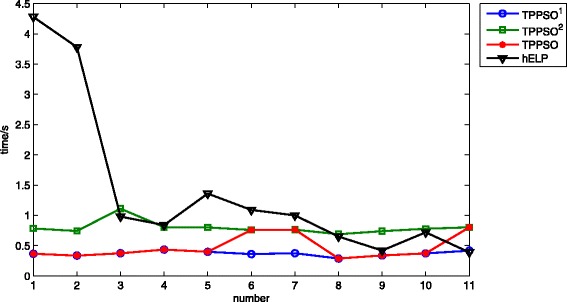
The average CPU time of our methods and hELP. The abscissa is the number of sequence, and the ordinate is CPU time. Because TPPSO^1^ can’t obtain the minimal free energy of sequence *A*_6_,*A*_7_ and *A*_11_, we chose smaller CUP time with minimal free energy for all sequences, denoted as TPPSO. In the figure, the CPU time of TPPSO^1^ and TPPSO^2^ are stable with respective optimal structure. CPU time of TPPSO also is stabler. But CPU time of hELP is fluctuant

The average CPU time of hELP was summarized in reference [14]. We also computed the average CPU time of TPPSO^1^ and TPPSO^2^. The average CPU time of all methods were shown in Fig. 6. It is obvious that the average CPU time of every sequence of TPPSO^1^ is the shortest, and that of TPPSO^2^ is longer, because TPPSO^2^ added the pulling strategy. For every sequence, the average CPU time of TPPSO^1^ and TPPSO^2^ are stable and vary around 0.4 and 0.8 s respectively. However, the average CPU time of hELP is not stable. Since TPPSO^1^ can’t obtain the lowest free energy of sequence *A*_6_, *A*_7_, and *A*_11_, we made TPPSO as the method which can fold the optimal structures of all sequences by PSO. The average CPU time of TPPSO was taken as less average CPU time of TPPSO^1^ and TPPSO^2^,which was also showed in Fig. 6. We know that the average CPU times of all sequences of TPPSO and hELP are 0.475 and 1.41 s respectively. It indicated that our method TPPSO is faster.

Table 5 summarized the number of H-H interactions and iteration number before the lowest free energy values are found by TPPSO^2^ (denoted as IBE number) for eleven sequences with 27 amino acids in the Table 3. It is obvious that the more H-H interactions, the more IBE numbers. The fitting function of the number of H-H interactions and IBE number was given as follows.

13 $$\begin{array}{*{20}l} & y=2307*e^{-\frac{(x-19.37)^{2}}{5.377^{2}}}+524.5*e^{-\frac{(x-3)^{2}}{1.904}} \end{array} $$

**Table 5:** IBE number of TPPSO ^2^

Sequence ID	*A*_8_	*A*_9_	*A*_3_	*A*_5_	*A*_1_	*A*_2_	*A*_10_	*A*_6_	*A*_7_	*A*_4_	*A*_11_
H-H ^a^	4	7	8	8	9	10	11	12	13	15	16
IBE number ^b^	3	14	44	61	177	439	83	812	805	19	2672

^a^H-H means the number of hydrophobic-hydrophobic amino acid interactions for optimal structure

^b^iteration numbers before the lowest free energy values are found

where *x* is the number of H-H pairs, and *y* is the IBE number.

**Fig. 7:**
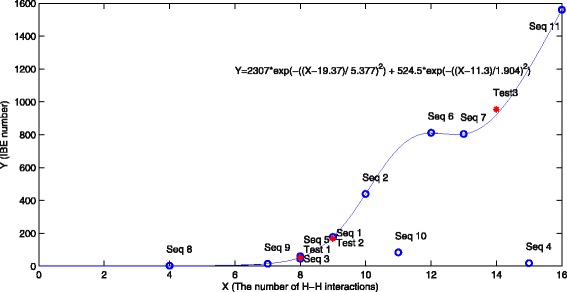
The fitting figure of IBE number and H-H interaction for sequences in table 1 by TPPSO^2^. The abscissa is H-H interaction of every sequence, and the ordinate is IBM number. Almost all sequences are satisfied with this fitting figure. IBE number will increase with H-H interaction for sequences with the same length. The three stars is the IBE number of test sequence with the same length 27. Their fitting function values are almost matched to computed IBE numbers

The figure of fitting function was exhibited in Fig. 7. Except for sequences *A*_4_ and *A*_10_, the IBE number of others are all close to the fitting function. The IBE number of sequence *A*_4_ and *A*_10_ are not satisfied with the fitting function, because in these sequences H amino acids and P amino acids are very dispersive, but in other sequences H segments or P segments are longer. We believed that IBE number of TPPSO^2^ is mainly affected by the number of H-H interactions for sequences with the same length. It tends to be larger with more H-H interactions. Moreover, the length of H or P segments will affect the IBE number.

In order to further verify the above conclusion, we simulated three test sequences with the same length (See Table 6). It is obvious that H segments and P segments of these test sequences are longer. IBE numbers for test sequences are close to the fitting curve (See Fig. 7) and all relative errors were showed in Table 6. It means that our inference about IBE number of TPPSO^2^ is reasonable.

**Table 6:** Test sequences

Sequence ID	Amino acids sequence	H-H	IBE number	Relative error
Test 1	*H*_4_ *P*_5_ *HP*_5_ *H*_3_ *P*_8_ *H*	8	51 (52.3829)	0.0271
Test 2	*H*_4_ *P*_5_ *HP*_5_ *H*_3_ *P*_4_ *HP*_3_ *H*	9	167 (177.8417)	0.0649
Test 3	(*HP*_2_ *HP*)_5_ *HP*	14	956 (921.1219)	0.0365

IBE number is the iteration number of every sequence by TPPSO^2^ before the minimal free energy was found. The number in parentheses is IBE number calculated by fitting function

#### Simulation of sequences with different length

**Fig. 8:**
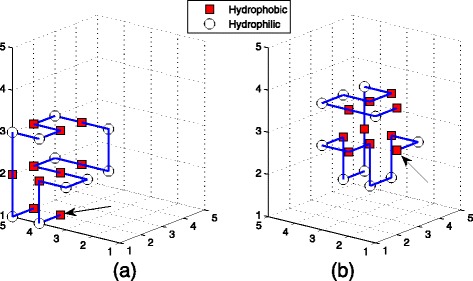
These figures are the structures of sequence B with 20 amino acids. **a** is one of structures by TPPSO^1^ with 11 H-H interactions. **b** is one of structures by TPPSO^2^ with 11 H-H interactions. By comparing, *left* structure is more compact, *right* structure is looser

We also computed several sequences with different length which have not been solved by EN and hELP. Moreover, Table 7 recorded the H-H interactions and IBE number of TPPSO^2^. These sequences were simulated by TPPSO^1^ and TPPSO^2^ respectively. The results of two methods are the same (See Table 7). They have the same H-H interactions. But we knows that the CPU time of TPPSO^2^ is shorter than one of TPPSO^1^, because TPPSO^2^ includes pulling strategy. For this reason, the structure obtained by TPPSO^2^ is more compact. It is illustrated in Fig. 8.

**Table 7:** Sequences with different lengths

Sequence ID	Amino acids sequence	Length	H-H	IBE number
B	*H*_4_ *P*_2_ *H*_7_ *P*_3_ *H*	17	9	2
C	*HPHP*_2_ *H*_2_ *PHP*_2_ *HPH*_2_ *P*_2_ *HPH*	20	11	11
D	*P*_2_ *HP*_2_ *H*_2_ *P*_4_ *H*_2_ *P*_4_ *H*_2_ *P*_4_ *H*_2_	25	9	139
E	*P*_3_ *H* ^2^ *P*_2_ *H*_2_ *P*_5_ *H*_7_ *P*_2_ *H*_2_ *P*_4_ *H*(*HP*_2_)_2_	36	17	432
F	*P*_2_ *H*(*P*_2_ *H*_2_)_2_ *P*_5_ *H*_10_ *P*_6_(*H*_2_ *P*_2_)_2_ *HP*_2_ *H*_5_	48	29	976

H-H interactions were the same by TPPSO^1^ and TPPSO^2^. IBE numbers were computed by TPPSO^2^. These sequences were simulated by different methods, and every method only folds a part of sequences. TPPSO^2^ found the minimal free energy of all sequences

These results shows that: a) TPPSO^2^ is able to solve sequences with different length and the obtained characteristic of protein structure is significant. b) pulling strategy improved the performance. c) Tabu search strategy avoided prematurity effectively. d) For TPPSO^2^, the longer the sequence, the more the IBE number.

### Probing protein stability upon amino acid mutation

Protein stability determines whether a protein will be in its native folded conformation or a denatured state. The folded, biologically active conformation of a protein is believed more stable than the unfolded, inactive conformations [15]. Thus, making proteins more stable is important in medicine and basic research. Amino acid mutations are widely used in protein design and analysis techniques to increase or decrease stability. These mutations are carried out experimentally using site-directed mutagenesis and similar techniques. This is time-consuming and often requires the use of computational prediction methods to select the best possible combinations [16–19]. With the efficient hybrid method at hand, we aim to probe the protein stability on 3D lattice. Particularly, we will simulate how single-site or double amino acid mutation affects protein stability. i.e., predicting the protein stability changes upon amino acid mutations with TPPSO^2^.

#### Single amino acid mutation

The hybrid method TPPSO^2^ has been tested to solve protein structure prediction problem. Now, we focused on single amino acid mutation, whether and which amino acid affects the stability of protein structure. The experiments is designed as follows. We firstly calculate the optimal H-H interactions of original sequence by TPPSO^2^. Then we choose one amino acid to mutate, i.e., we change it from H (P) into P (H). Then we calculate the optimal H-H interactions of mutated sequence by TPPSO^2^. Finally the deviation of H-H interactions between mutated sequence and original sequence was recorded.

##### Sequences with different length

In order to probe the stability of amino acid mutation, we chose four sequences with different lengths. These sequences were mentioned in the above section. They are sequence *B*, *C*, *D* and *A*_8_.

**Fig. 9:**
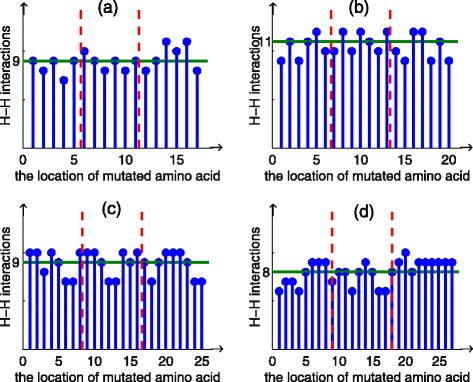
These figures are H-H interactions of sequences after single amino acid mutation. The abscissa is the location of mutated amino acid, and the ordinate is the number of H-H interaction for mutated sequence. The *horizonal line* is the number of H-H interaction of original sequence. Two vertical lines spit sequence into three equal parts. **a** is mutational results of sequence B. In this figure, 100% pivotal amino acids locate at beginning or ending of sequence B. **b** is mutational results of sequence C. In this figure, 100% pivotal amino acids locate at beginning or ending of sequence C. **c** is mutational results of sequence D. In this figure, 71.4% pivotal amino acids locate at beginning or ending of sequence D. **d** is mutational results of sequence *A*_8_. In this figure, 50% pivotal amino acids locate at beginning or ending of sequence *A*_8_

Figure 9 recorded the H-H interactions of every single amino acid mutational sequence. From the results, we found that some mutational amino acids will result in a bigger deviation. We call those pivotal amino acids. The ratios of pivotal amino acids are 100%, 100%, 71.4% and 50% respectively for the four sequences. Also we deduced that those pivotal amino acids tend to locate at the beginning or end of sequence.

Tables 8, 9, 10 and 11 recorded the characters of mutated sequences including the deviation between mutated sequence and original sequence, the quantity of amino acid which mutated to cause the deviation and the ratio of every deviation. According to the results, we found that single amino acid mutation has the maximal and minimal deviation 2 and -2. The results also indicated that the ratio of maximal deviation is around 22%.

**Table 8:** The single amino acid mutation results for sequence B

D-value	-3	-2	-1	0	1	2	3
Q-value	0	1	5	7	2	2	0
R-value	0%	**6%**	2%	41%	12%	**12%**	0%

There are 9 H-H interactions by TPPSO^2^ for original sequence B. Every amino acid would be mutational, namely H (P) was changed into P (H). D-value is the deviation of H-H interactions between new sequence and original sequence when single amino acid was mutated. Q-value is the number of amino acids caused the deviation. R-value is the ratio of amino acids. The ratio of amino acids which caused the maximal deviation is 18% (summarization of the numbers highlighted in bold)

**Table 9:** The single amino acid mutation results for sequence C

D-value	-3	-2	-1	0	1	2	3
Q-value	0	5	5	4	6	0	0
R-value	0%	**25%**	25%	20%	30%	**0%**	0%

There are 11 H-H interactions by TPPSO^2^ for original sequence C. Every amino acid would be mutational, namely H (P) was changed into P (H). The ratio of amino acids which caused the maximal deviation is 25% (summarization of the numbers highlighted in bold)

**Table 10:** The single amino acid mutation results for sequence D

D-value	-3	-2	-1	0	1	2	3
Q-value	0	7	1	6	11	0	0
R-value	0%	**28%**	4%	24%	44%	0%	0%

There are 9 H-H interactions by TPPSO^2^ for original sequence D. Every amino acid would be mutational, namely H (P) was changed into P (H). The ratio of amino acids which caused the maximal deviation is 28% (summarization of the numbers highlighted in bold)

**Table 11:** The single amino acid mutation results for sequence *A*_8_

D-value	-3	-2	-1	0	1	2	3
Q-value	0	5	3	7	11	1	0
R-value	0%	**19%**	11%	26%	41%	**3%**	0%

There are 8 H-H interactions by TPPSO^2^ for original sequence *A*_8_. Every amino acid would be mutational, namely H (P) was changed into P (H). The ratio of amino acids which caused the maximal deviation is 22% (summarization of the numbers highlighted in bold)

Table 12 summarized the effect of hydrophobic (hydrophilic) amino acid mutation on H-H interactions. We know that hydrophobic amino acid mutation would not make the H-H interactions increase and hydrophilic amino acid mutation would not lead the H-H interactions decrease. It means that hydrophilic amino acid mutation will result in more compact structure, while hydrophobic amino acid mutation will result in the looser structure. By comparing the results of H^2^ and P^2^ in the Table 12, we suppose that hydrophobic amino acid is more impressible than hydrophilic amino acid to reflect stability of protein structure for sequence with different lengths.

**Table 12:** Summary of the single mutation results

Sequence ID	H^0^	P^0^	H^1^	P^1^	H^2^	P^2^
B	12	5	12	5	1	2
C	10	10	10	10	5	0
D	9	9	16	16	7	0
*A*_8_	6	21	6	21	5	1

H^0^ and P^0^ are the number of hydrophobic and hydrophilic in the original sequence. H^1^ is the number of mutational hydrophobic amino acid whose H-H interactions is not more than the original one. P^1^ is the number of mutational hydrophilic amino acid whose H-H interactions is not less than the original one. H^2^ is the number of hydrophobic amino acid which caused the maximal deviation with original H-H pairs. P^2^ is the number of hydrophilic amino acid which caused the maximal deviation with original H-H pairs

**Fig. 10:**
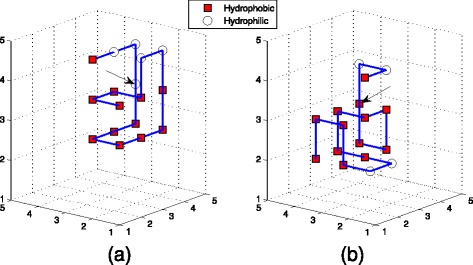
These figures are the structures of sequence B before mutating and after mutating respectively. **a** is the optimal structure with 9 H-H interactions predicted for original sequence B by TPPSO^2^. **b** is one of optimal structures predicted for mutated sequence B by TPPSO^2^, in which the 14th amino acid was mutated. The mutated amino acid is denoted by *arrow* in figures. Since the 14th amino acid was changed from P to H, the number of H-H interaction increases and the deviation is 2. It is obvious that right structure is more compact

The structures of sequence B before and after mutation are showed in Fig. 10. Since the 14th amino acid was changed from P to H, the number of H-H interaction increases and the deviation is 2, which is the maximal deviation. It is obvious that optimal structures of 14th amino acid mutation is more compact.

**Fig. 11:**
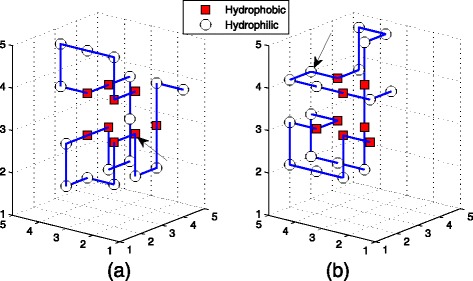
These figures are the structures of sequence D before mutating and after mutating respectively. **a** is the optimal structure with 9 H-H interactions predicted for original sequence B by TPPSO^2^. **b** is one of optimal structures predicted for mutated sequence C by TPPSO^2^, in which the 6th amino acid was mutated. The mutated amino acid is denoted by *arrow* in figures. Since the 6th amino acid was changed from H to P, the number of H-H interaction decreases and the deviation is 2. It is obvious that left structure is more compact

The structures of sequence D before and after mutation are showed in Fig. 11. Since the 6th amino acid was changed from H to P, the number of H-H interaction decreases and the deviation is 2 which is the maximal deviation. It is obvious that optimal structure of original sequence is more compact.

##### Sequences with the same length

We selected five sequences from Table 3 to test what kind of protein structures are more stable upon single amino acid mutation by TPPSO^2^. We changed every amino acid of these sequences, then recalculated and recorded the H-H interactions of every mutated sequence.

Table 13 showed that: 1) For the sequences with the same length, UC number is likely larger with more mass number. The exception might be caused by the fact that UC number reflected the stability of protein structure. Namely, sequence with larger UC number was susceptible to single amino acid mutation. 2) According to H-H interactions and the number of hydrophobic amino acids, the sequence with more hydrophobic amino acid usually had more H-H interactions. 3) From P →H and H →P, most of mutational hydrophobic amino acids made the H-H interactions changed. Relatively, only a part of hydrophilic amino acids affect the number of H-H interactions. We conclude that hydrophobic amino acid is more impressible than hydrophilic amino acid to reflect stability of protein structure for sequence with the same length.

**Table 13:** Single amino acid mutation of sequences with 27 amino acids

Sequence ID	H-H	mass number	UC number	H	P	P →H	H →P
*A*_8_	4	9	4	6	21	19	4
*A*_3_	8	7	2	9	18	17	8
*A*_5_	8	8	7	9	18	12	8
*A*_10_	11	17	7	11	16	9	11
*A*_4_	15	13	10	14	13	3	14

H-H is the number of H-H pairs of original sequence. The same continuous amino acids were taken as a mass. Mass number is for original sequence. UC number is the number of mutational amino acid which does not change the H-H interactions. H (P) is the number of hydrophobic (hydrophilic) amino acids in the original sequence. P →H (H →P) is the number of mutational hydrophilic (hydrophobic) amino acid which affected the H-H interactions

All these results illustrated that: a) the more hydrophobic amino acids, the more H-H interactions; b) sequence with more H-H interactions tends to be more stable when single amino acid is mutated; c) hydrophobic amino acid mutation tends to alter the protein structure largely.

According to the above observations, we summarize that the sequence with more hydrophobic amino acids will be less susceptible to single amino acid mutation.

#### Double neighbouring amino acids mutation

Amino acid does not work alone and multiple amino acids coordinate to maintain stability and perform function. Our in-silicon simulation allows us to go beyond single amino acid mutation and explore the combinatorial effect of amino acid mutation. In this section, we explore the effect of double neighbouring amino acids mutation (two adjacent amino acids are mutated) in protein folding. Double neighbouring amino acids mutations were classified as HH → PP, PP → HH, HP → PH, PH → HP.

We simulated three sequences (B, C, D) with different length when adjacent amino acids mutated. The maximal deviation and locations of pivotal amino acids were conserved (See Tables 14, 15, 16).

**Table 14:** Double amino acids mutation results for sequence B

D-vale	HH → PP (9) ^a^	PP → HH (3) ^a^	HP → PH (2) ^a^	PH → HP (2) ^a^
-2	4(H,M,T) ^b^	0	0	0
-1	5	0	1	0
0	0	0	0	0
+1	0	1	1	1
+2	0	2(T)	0	1(T)

^a^The number in parentheses is the number of adjacent amino acids in sequences. D-value is the deviation of minimal free energy caused by neighboring mutational amino acids

^b^The position of mutational double amino acids. H (M,T) means that the mutational double amino acids is at head (middle, tail) of the sequence

**Table 15:** Double amino acids mutation results for sequence C

D-vale	HH → PP (2)	PP → HH (3)	HP → PH (7)	PH → HP (7)
-3	1(H)	0	0	0
-2	1(T)	0	0	0
-1	0	0	3	3
0	0	0	4	4
+1	0	2	0	0
+2	0	1(H)	0	0

**Table 16:** Double amino acids mutation results for sequence D

D-vale	HH → PP (4)	PP → HH (11)	HP → PH (4)	PH → HP (5)
-2	4(H,M,T)	0	0	0
-1	0	0	0	3
0	0	0	2	1
+1	0	5	1	1
+2	0	6(M,T)	1(T)	0

Tables 14, 15 and 16 recorded the variation of H-H interactions and the position of pivotal double amino acids. According to these tables, we concluded that: a) If double amino acids mutation was HH → PP or PP → HH, the H-H interactions must be changed. But PH →HP and HP →PH maybe have variation. b)HH → PP and PP → HH must make the H-H interactions decrease and increase, respectively. c) The effect of double adjacent amino acids mutation which belongs to HP → PH or PH → HP was finite. d) The position of pivotal double adjacent amino acids mutation tend to locate be at the head or tail of sequence.

#### Double arbitrary amino acids mutation

We continued to explore the combinatorial effect of amino acid mutation. In this section, we check the effect of double amino acids mutations with arbitrary distance in protein folding. The amino acid mutations were classified ed as HH → PP, PP → HH, HP → PH, PH → HP. We simulated the sequence B in Table 7 with 20 amino acids. There are 10 hydrophobic amino acids and 10 hydrophilic amino acids in sequence B. We folded the conformations of all of mutation sequences.

Form Table 17, we knew that combinations of H+H and P+P are more sensitive and easier to affect the stability of protein structure. We simulated every mutational sequence. The results showed that a) all HH →PP mutations will decrease HH interactions, namely HH interactions won’t increase; b) 43 PP →HH mutations will increase HH interactions, 2 PP →HH mutations won’t change HH interactions, in other words, HH interactions won’t decrease; c) HP →PH or PH →HP mutations will not influence HH interactions.

**Table 17:** Double arbitrary amino acids mutation results for sequence B

Combination	O-num	V-num	V-rate
H+H	45	45(*↓*)	100%
P+P	45	43(*↑*)	96%
H+P	50	29(*↑* *↓*)	58%
P+H	50	18(*↑* *↓*)	38%

H+H(P+P) means that arbitrary double hydrophobic(hydrophilic) amino acids will be mutated. H+P(P+H) means that hydrophobic(hydrophilic) will match with hydrophilic(hydrophobic) behind of it to mutate. O-num is original combination number in sequence. V-num is the number of combinations with which minimal free energy were altered after mutating. V-rate is the rate of V-num. The arrows in parentheses indicate increase or decrease of the free energy

The simulation results in Table 18 indicated that **a)** closer H and H (or P and P) can result in D-value; **b)** amino acid *H*_18_ respectively matches amino acid *H*_20_ and amino acid *P*_10_ to obtain maximal deviation of structure, so amino acid *H*_18_ is the most sensitive amino acid, which is at the tail of sequence; **c)** amino acid *H*_3_ will cause D-value with hydrophilic (P), so amino acid *H*_3_ is very sensitive to polar, it is at the head of sequence; **d)** matching amino acid *H*_7_ with arbitrary amino acid P, HH interactions are invariable, so *H*_7_ is obtuse, but by combining *H*_7_ with arbitrary H, it is sensitive, this amino acid is in the middle of sequence; **e)** *H*_1_ and *H*_20_ are impressible for other H, but they are stable for arbitrary P, the mutations of *H*_1_ and *H*_20_ with all of P lead to decrease one more HH interaction, *H*_1_ and *H*_20_ are at the head and tail of sequence, respectively.

**Table 18:** Combination D-value and pivotal amino acids results for sequence B

Combination D-value and pivotal amino acids		
H+H	-4	*H*_1_ *H*_3_,*H*_18_ *H*_20_
P+P	+2	*P*_4_ *P*_5_,*P*_4_ *P*_13_,*P*_5_ *P*_8_,*P*_8_ *P*_17_,*P*_10_ *P*_13_,*P*_11_ *P*_16_,*P*_13_ *P*_16_,*P*_16_ *P*_19_
H+P	-2	*H*_3_ *P*_10_,*H*_3_ *P*_16_,*H*_3_ *P*_19_,*H*_6_ *P*_19_
P+H	-3	*P*_10_ *H*_18_

H+H(P+P) means that arbitrary double hydrophobic(hydrophilic) amino acids will mutate. H+P(P+H) means that hydrophobic(hydrophilic) will match with hydrophilic(hydrophobic) behind of it to mutate. D-value is the maximal deviation of H-H interactions between new sequence and original sequence when double arbitrary amino acids mutation. *H*_*i*_ *H*_*j*_ means that the *i* ^*th*^ animo acid matches the *j* ^*th*^ amino acid to mutate, in which two mutational amino acids are hydrophobic

According to the above observations, we summarized that a) double arbitrary amino acids mutation will be more sensitive to affect protein stability; b) double amino acids mutation with the same hydrophilic or hydrophobic property is more unstable than double amino acids mutation with different property; c) most of sensitive combinations are at the head or tail of sequence.

## Discussion

As many research results indicate, HP model is very useful for modelling protein properties though it is simple and has many disadvantages. It captures the main difficulty of the real world problem. HP model has been applied in investigation of ligand binding to proteins [20]. The distinct influences of function, folding, and structure on the evolution of HP model are studied, by exhaustive enumeration of conformation and sequence space on a two dimensional lattice, which costs four week’s computation [21]. These research all show that our effort to fold the HP chain by a hybrid method on 3D lattice is necessary and important.

Also we propose to use HP model to probe the protein stability. HP model serves as a very efficient tool here. The simplification of 20 amino acids to H, and P types dramatically reduce the possible mutation pattern. Especially we can easily perform the double mutation only considering four combinations. Those insights from the HP model can serve as novel hypothesis to guide experiments. We also need to point out that the protein stability results and conclusions are heavily depending on the optimal solution of 3D HP model. We demonstrate the results in some small scale problems. When we want to generalize the study, we need to further improve the hybrid algorithm.

In our study, the computational experiments show that the new hybrid algorithm is efficient for short sequences. When the input space is bigger, there will be some sub-optimal solutions and more difficult to find the minimal energy configurations. It’s really a challenge for large scale HP model. The conformation space grows rapidly as the chain length increases. A possible method is to introduce divide-and-conquer strategy. We can also consider to combine with other algorithms or start from a good initial point from biological view. It will be our future work in devising such an algorithm for large protein.

## Conclusion

In this paper, we studied protein structure prediction problem on 3D square lattice. We summarize the findings of this work as follows. Firstly, we formulated the protein structure prediction problem on 3D lattice into a combinatorial optimization problem; secondly, basic PSO algorithm has been enhanced to deal with discrete optimization problem; thirdly, we proposed a novel hybrid method (TPPSO^2^) and proved its feasibility by simulating; fourthly, we derived some interesting insights for protein stability via single and double amino acid mutation perturbation.
